# Transcriptomic responses of oil palm (*Elaeis guineensis*) stem to waterlogging at plantation in relation to precipitation seasonality

**DOI:** 10.3389/fpls.2023.1213496

**Published:** 2023-08-10

**Authors:** Hui Lim, Masaki J. Kobayashi, Sri Nugroho Marsoem, Denny Irawati, Akihiko Kosugi, Toshiaki Kondo, Naoki Tani

**Affiliations:** ^1^ Graduate School of Science and Technology, University of Tsukuba, Tsukuba, Ibaraki, Japan; ^2^ Forestry Division, Japan International Research Center for Agricultural Sciences (JIRCAS), Tsukuba, Ibaraki, Japan; ^3^ Faculty of Forestry, Universitas Gadjah Mada (UGM), Yogyakarta, Indonesia; ^4^ Biological Resources and Post-harvest Division, Japan International Research Center for Agricultural Sciences (JIRCAS), Tsukuba, Ibaraki, Japan; ^5^ Faculty of Life and Environmental Sciences, University of Tsukuba, Tsukuba, Ibaraki, Japan

**Keywords:** waterlogging, *Elaeis guineensis*, transcriptomic, climate change, transcription factors

## Abstract

Global warming-induced climate change causes significant agricultural problems by increasing the incidence of drought and flooding events. Waterlogging is an inevitable consequence of these changes but its effects on oil palms have received little attention and are poorly understood. Recent waterlogging studies have focused on oil palm seedlings, with particular emphasis on phenology. However, the transcriptomic waterlogging response of mature oil palms remains elusive in real environments. We therefore investigated transcriptomic changes over time in adult oil palms at plantations over a two-year period with pronounced seasonal variation in precipitation. A significant transcriptional waterlogging response was observed in the oil palm stem core but not in leaf samples when gene expression was correlated with cumulative precipitation over two-day periods. Pathways and processes upregulated or enriched in the stem core response included hypoxia, ethylene signaling, and carbon metabolism. Post-waterlogging recovery in oil palms was found to be associated with responses to heat stress and carotenoid biosynthesis. Nineteen transcription factors (TFs) potentially involved in the waterlogging response of mature oil palms were also identified. These data provide new insights into the transcriptomic responses of planted oil palms to waterlogging and offer valuable guidance on the sensitivity of oil palm plantations to future climate changes.

## 1 Introduction

Agricultural crops, and particularly cereals, are vital components of human diets and have therefore been studied extensively (Parent et al., 2008; Loreti and Perata, 2020). Erratic climate change has increased the frequency and severity of crop damage caused by abiotic stresses such as extreme drought, flooding, and temperatures. This has provoked worldwide interest in research aiming to increase the climate resilience of agricultural crops, and climate resilience has become a major additional target of genetic engineering and breeding efforts. One of the many environmental changes caused by climate change is the waterlogging of agricultural crops due to rapid rainfall. Waterlogging effects caused by extreme rainfall have been studied extensively in other agronomic crops and tree species, and are known to cause drastic yield loss and growth retardation (Glenz et al., 2006; Kreuzwieser and Rennenberg, 2014; Miyazawa et al., 2014; Tian et al., 2021; Langan et al., 2022). The situation is similar in oil palm cultivation; many studies have shown that oil palms are very sensitive to erratic climate change (Corley and Tinker, 2015; Paterson and Lima, 2018; Abubakar et al., 2021). This has exacerbated the pressure on the oil palm industry caused by oil palm fungus attacks, which can reduce yields by 50-80%: extreme rainfall usually increases environmental humidity, which favors fungal growth (Paterson et al., 2013; Corley and Tinker, 2015; Torres et al., 2016; Paterson and Lima, 2018; Abubakar et al., 2021). Oil palm yield loss is of particular concern because the oil palm has become a major cash crop in several countries, especially Indonesia and Malaysia. Nevertheless, waterlogging-response study of oil palm is still at its rudimentary stage and apparently investigated on oil palm seedlings grown under controlled conditions (Rivera-Mendes et al., 2016; Cui et al., 2019; Nuanlaong et al., 2020; Nuanlaong et al., 2021); there have been no field studies on waterlogging responses in oil palms growing in real environments. Sessile plants have evolved complex response mechanisms that let them adapt to and survive environmental fluctuations that are not readily reproduced in controlled experiments. Additionally, adult oil palms are substantially larger and more advanced in their development than seedlings, which might make them more resistant to waterlogging but also makes it difficult to study them in controlled experiments (Rankenberg et al., 2021). Finally, field studies are important because they can provide direct information on impacts of climatic change that cannot be studied under controlled experimental conditions. As such, they are vital for verifying the results of controlled experiments.

The waterlogging responses under field environment could be addressed using omics methods such as genomics, transcriptomics, and proteomics, all of which have been used extensively to study plants’ molecular responses to climate change. In particular, a transcriptomic differential gene expression analysis could reveal waterlogging-responsive genes in planted oil palms and thereby clarify their molecular responses to waterlogging. A recent transcriptomic study on the drought stress response in oil palms successfully identified thousands of differentially expressed genes (DEGs) in palm seedling roots (Wang et al., 2020), suggesting that a similar approach could be used to identify DEGs related to waterlogging responses.

To the best of our knowledge, the underground soil environment differs markedly from the aboveground atmospheric environment, particularly in terms of the availability of oxygen and water. Roots were proclaimed to be the very first part of plant system to discern water-deficiency or inundation, which then transmit various molecular signals to other parts of the plant via long-distance communication (Takahashi et al., 2020; Yang et al., 2020). Consequently, most previous studies on waterlogging have focused primarily on the roots and rarely scrutinized into waterlogging responses of other plant parts (Rivera-Mendes et al., 2016; Nuanlaong et al., 2020; Nuanlaong et al., 2021). Leaves may respond strongly to waterlogging because they are highly sensitive to osmotic stress and water deficiency in the atmosphere, and leaf morphological traits are well-established indicators of plant water status. For instance, leaves exhibit highly variable gene expression under drought stress and are sensitive to environmental variations (Kreuzwieser et al., 2009; Carr, 2011; Cui et al., 2019). It would also be interesting to investigate waterlogging responses in the oil palm trunk (i.e., the stem) because the trunk is the largest part of an oil palm tree and serves as the plant’s main carbohydrate sink (Legros et al., 2009; Tani et al., 2020). Moreover, an earlier study on citrus showed that trunk growth depends strongly on the plant’s water status (Pérez-Jiménez and Pérez-Tornero, 2021), indicating that trunk-level homeostatic responses may exist. The effects of waterlogging on plant trunks are poorly understood because they have rarely been studied. However, it is clear that waterlogging responses do not occur only in plants’ roots and may differ between parts of the plant, giving rise to tissue- or organ-specific expression patterns (Ellis et al., 1999; Kreuzwieser et al., 2009; Mustroph et al., 2014; Lothier et al., 2020).

This study was initiated to clarify waterlogging molecular responses at the transcriptomic level in the non-model crop species *Elaeis guineensis* (oil palm) by identifying waterlogging-related DEGs, biological processes, and regulatory networks. The results obtained reveal potential waterlogging-responsive traits that could be targeted for genetic modification in order to create climate-resilient oil palm varieties and help secure the future health of the oil palm industry.

## 2 Materials and methods

### 2.1 Meteorological data

Daily climate data for the Lampung study site were acquired from a meteorological station located at Radin Inten II (5°14'23"S 105°10'27"E), approximately 39 km away from the study site (data provided by the Indonesian meteorological department). During the studied period, the lowest and highest average temperatures at the palm plantation were 24 °C and 30 °C, respectively. The meteorological data show that Lampung had a tropical climate with an average annual precipitation of approximately 1741 mm during the experimental period. However, there were several months with extremely low or high precipitation levels.

### 2.2 Field sampling

This study was conducted at a flatland palm plantation (formerly a rubber plantation) in Lampung on the southern tip of Sumatera Island in Indonesia (5°16'59.8"S, 105°19'56.1"E). Two healthy adult oil palm trees with the almost same size (ca. 50 cm of stem diameter) being about 20 meters apart from each other were chosen as study subjects from a group of 19-21 year-old oil palm trees (tenera cultivars) that had remained undisturbed in the plantation environment because the trees underwent no fruit pruning, defoliation, and fertilization during the experimental period.

Time series sampling was conducted at nine time points from 2017 to 2019: 22 August 2017 (T1), 13 November 2017 (T2), 24 February 2018 (T3), 29 May 2018 (T4), 24 August 2018 (T5), 20 November 2018 (T6), 28 February 2019 (T7), 28 May 2019 (T8), and 7 September 2019 (T9). Stem cores and leaves were collected from the two selected oil palms around the noon. A Haglof increment borer (5.15 mm diameter) was used to extract stem core samples from palm tree trunks approximately 1 m below the branch of the lowest frond. For leaf sampling, we selected leaves from the middle part of mature palm fronds and cut them into small pieces. The collected plant materials were immediately placed into a 5 mL plastic tube containing RNAprotect Tissue Reagent solution (Qiagen, Germany) and kept at -20 °C at Universitas Gadjah Mada’s laboratory before being transferred to the laboratory at the Japan International Research Center for Agricultural Sciences (JIRCAS) where samples were stored at -80 °C before RNA extraction. A schematic diagram showing sampling procedure of this study presented in **Figure S1**.

### 2.3 RNA-seq library construction

Total RNA extraction was performed using approximately 50 mg plant material with the RNeasy® Plus Mini Kit (Qiagen, Germany) following the manufacturers’ guidelines. The quality and quantity of extracted total RNAs were determined using a NanoDrop^TM^ One-C (ThermoFisher Scientific, US), Qubit fluorometer (ThermoFisher Scientific, US) and an Agilent 4150 TapeStation system (Agilent, US) according to the manufacturers’ protocols before being sent for sequencing. A cDNA library of core samples was generated by preparing the extracted total RNA with the TruSeq Stranded mRNA LT Sample Prep Kit (Illumina, US) before performing paired-end sequencing (150 bp) with the NovaSeq 6000 platform to obtain 40 million reads of raw data (6 GB per sample) (Macrogen, South Korea). For leaf samples, a cDNA library was prepared with the NEBNext® UltraTM RNA Library Prep Kit (New England Biolabs, US) and sequenced in the same way by Novogene (Novogene Co. Ltd., China).

### 2.4 Identification of differentially expressed genes

The sequencing reads were pre-processed with Trimmomatic-0.39 (Bolger et al., 2014) to trim low-quality sequences and sequencing adapters. Using Hisat2 (ver. 2.2.1), trimmed reads were then mapped to a representative oil palm genome (Singh et al., 2013) downloaded from the NCBI database as described by (Pertea et al., 2016). The number of mapped reads was quantified with Stringtie (ver. 2.1.7) and read count data were obtained. The count data for the stem core and leaf samples were standardized separately using the TMM method as implemented in the R package edgeR (ver. 3.36.0) (Robinson et al., 2010). Each set of standardized count data was then analysed for DEGs associated with the Lampung precipitation data by performing likelihood ratio tests (glmLRT) using edgeR with a Benjamini-Hochberg false discovery rate (FDR) of 0.05 (Benjamini and Hochberg, 1995). To determine how to aggregation of the precipitation data affected the number of identified DEGs, we performed DEG analyses in which gene expression was related to cumulative precipitation over a number of days ranging from 1 to 10. The expression data were processed by scaling the log-transformed counts per million (CPM) for the DEGs such that they had a mean of zero and a standard deviation of one. A gene expression heatmap was then generated with the R package pheatmap (ver. 1.0.12) using the “ward.D” clustering method. To increase biological replicates, we make two different groups which representing waterlogging and non-waterlogging conditions. The alternative approach by grouping T3 and T7 expression data as waterlogging group and then compared to non-waterlogging group consisting of expression data from other sampling time points (Waterlogging grouping comparison). The gene expression analysis procedure and heatmap generation were same as described earlier. Simple overlapping between PDEGs/NDEGs from both approaches was conducted to obtain overlapped DEGs. A similarity score was acquired by referring to proportion of number of overlapped PDEGs/NDEGs to the number of PDEGs/NDEGs from former approach. 

### 2.5 Functional analysis of DEGs

To understand the functions of the DEGs, we annotated the *E. guineensis* genes using data for the corresponding *Arabidopsis thaliana* genes, which were identified based on sequence homology by performing BLASTP searches (E-value cutoff: 1.0E–10) (Altschul et al., 1990). Having identified the corresponding *A. thaliana* genes, we first investigated whether the set of DEGs contained a significant number of waterlogging-related genes by comparing them to a list of genes obtained from an *A. thaliana* submergence experiment (Yeung et al., 2018). Fisher’s exact test with the Bonferroni multiple testing correction was used to evaluate the significance of associations between overlapping DEGs from *E. guineensis* and *A. thaliana*, with a *p*-value threshold of 0.05 after applying the Bonferroni correction. To identify biological processes (BP) and pathways associated with the DEGs, we conducted Gene Ontology (GO) and Kyoto Encyclopedia of Genes and Genomes (KEGG) enrichment tests with a *p*-value threshold of 0.05 using the clusterProfiler package (ver. 4.2.2) available in R (Ashburner et al., 2000; Kanehisa and Goto, 2000). Gene ontology (GO) data for each *E. guineensis* gene were obtained by retrieving GO information for the corresponding *A. thaliana* gene from The Arabidopsis Information Resource (TAIR) (Swarbreck et al., 2008). The overlapped DEGs obtained through overlapping of PDEGs/NDEGs from both approaches also further enriched by GO and KEGG enrichment tests. The workflow of processing RNA-seq data in this study was summarized in **Figure S2**.

### 2.6 Gene regulatory network analysis for DEGs

To elucidate the transcriptional regulatory relationships between DEGs, we carried out a gene regulatory network (GRN) analysis using the GENIE3 package (ver. 1.16.0) in R. For this analysis, we obtained information on the transcription factors (TFs) of *A. thaliana* from PlantTFDB v5.0 (http://planttfdb.gao-lab.org) and identified potential TFs in *E. guineensis* based on homology with *A. thaliana* genes.

## 3 Results

### 3.1 Seasonal patterns of precipitation at the Lampung oil palm plantation

**Figure 1:**
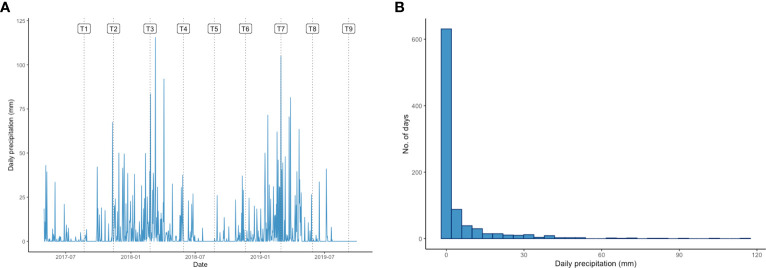
**(A)** Daily precipitation (mm) pattern in Lampung over the experimental period. Labels T1-T9 indicate the sampling time points for this study: 22 August 2017 (T1), 13 November 2017 (T2), 24 February 2018 (T3), 29 May 2018 (T4), 24 August 2018 (T5), 20 November 2018 (T6), 28 February 2019 (T7), 28 May 2019 (T8), and 7 September 2019 (T9). **(B)** Histogram showing the daily precipitation distribution (mm) in Lampung during the experimental period.

The daily precipitation at the plantation over the experimental period labelled with T1-T9 which indicating the sampling dates is shown in **Figure 1**. During the studied period, the precipitation around the T3 and T7 sampling points was clearly significantly more intense than at any other sampling point. The average daily precipitation in Lampung was 4.73 mm and the highest daily precipitation recorded during the study was 115.5 mm. Despite the daily precipitation was generally around 0-4 mm, the histogram presented in **Figure 1** shows that it was above 30 mm on approximately 5 % of the days during the experimental period. This level was considered indicative of heavy rain that could cause oil palm waterlogging (**Figure 1**).

### 3.2 Identification of DEGS correlating with precipitation in oil palms

**Figure 2:**
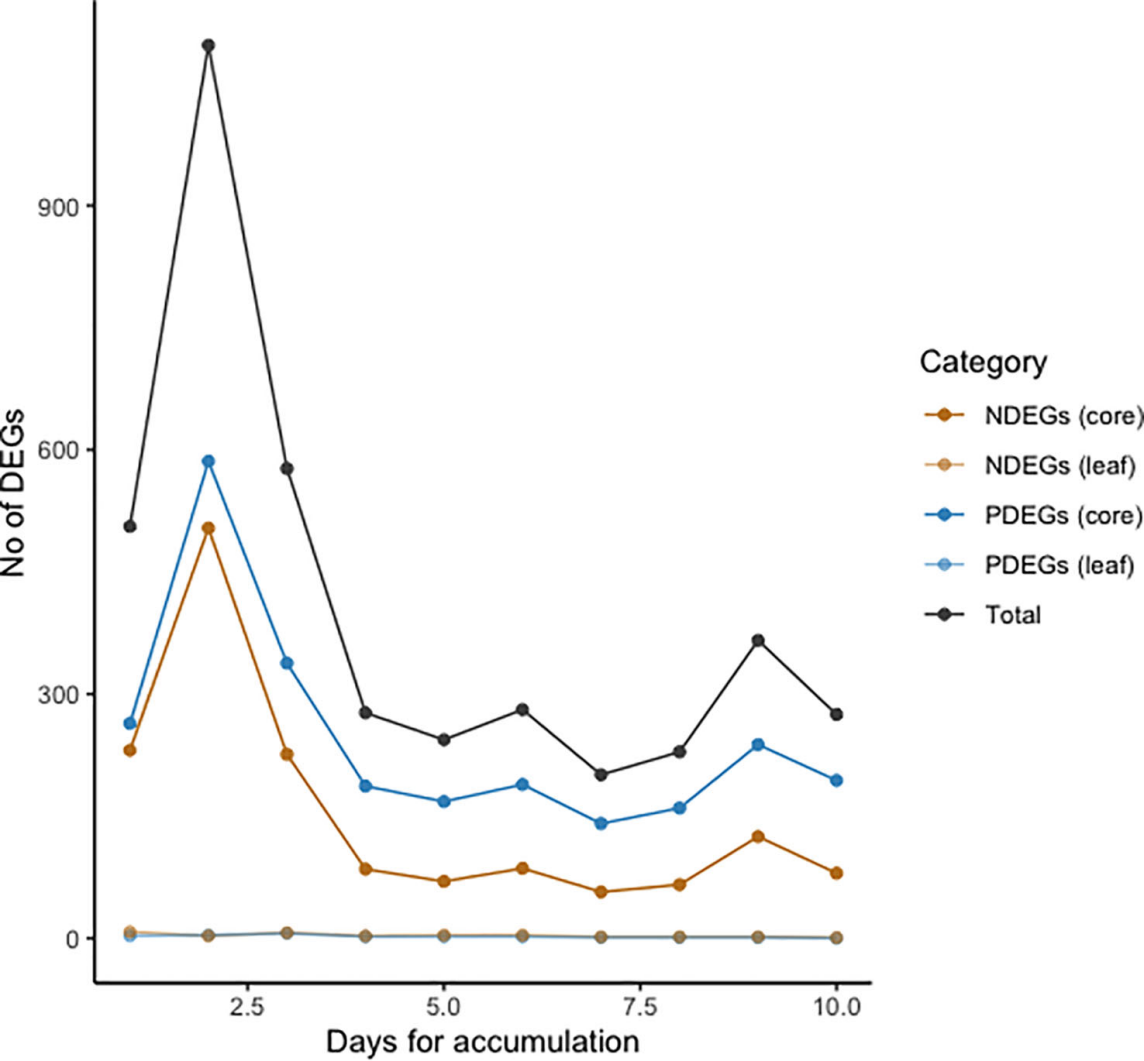
Numbers of positively correlated differentially expressed genes (PDEGs) (blue line), negatively correlated DEGs (NDEGs) (orange line) and total DEGs (black line) in stem core and leaf samples from mature oil palms. DEGs were identified by analysing the relationship between gene expression and cumulative precipitation intensity at Lampung over periods of 1-10 days.

To determine whether heavy rain changes gene expression in oil palms, we tried to identify genes responding to rainfall in the stem core and leaf samples. However, it was not clear how the magnitude of the oil palm response would depend on the cumulative precipitation over multiple days. We therefore first investigated how the number of identified DEGs depended on the number of days over which precipitation was aggregated in the DEG analysis, which was varied from 1 to 10. The number of detected DEGs peaked when precipitation was aggregated over two days for the stem core and three days for leaf samples (**Figure 2**). Additionally, the number of DEGs in stem cores greatly exceeded that in leaves: we identified 586 positively correlated DEGs (PDEGs) and 504 negatively correlated DEGs (NDEGs) in the stem core with precipitation aggregated over two days (**Figure S3**). Conversely, we identified only 6 PDEGs and 7 NDEGs in leaves with precipitation aggregated over three days (**Figure S4**). Because this analysis suggested that stem cores responded more strongly to rainfall than the leaves, all of our subsequent analyses focused on the stem core DEGs, which were identified based on the correlation between their expression and the cumulative precipitation over two-day periods.

**Figure 3:**
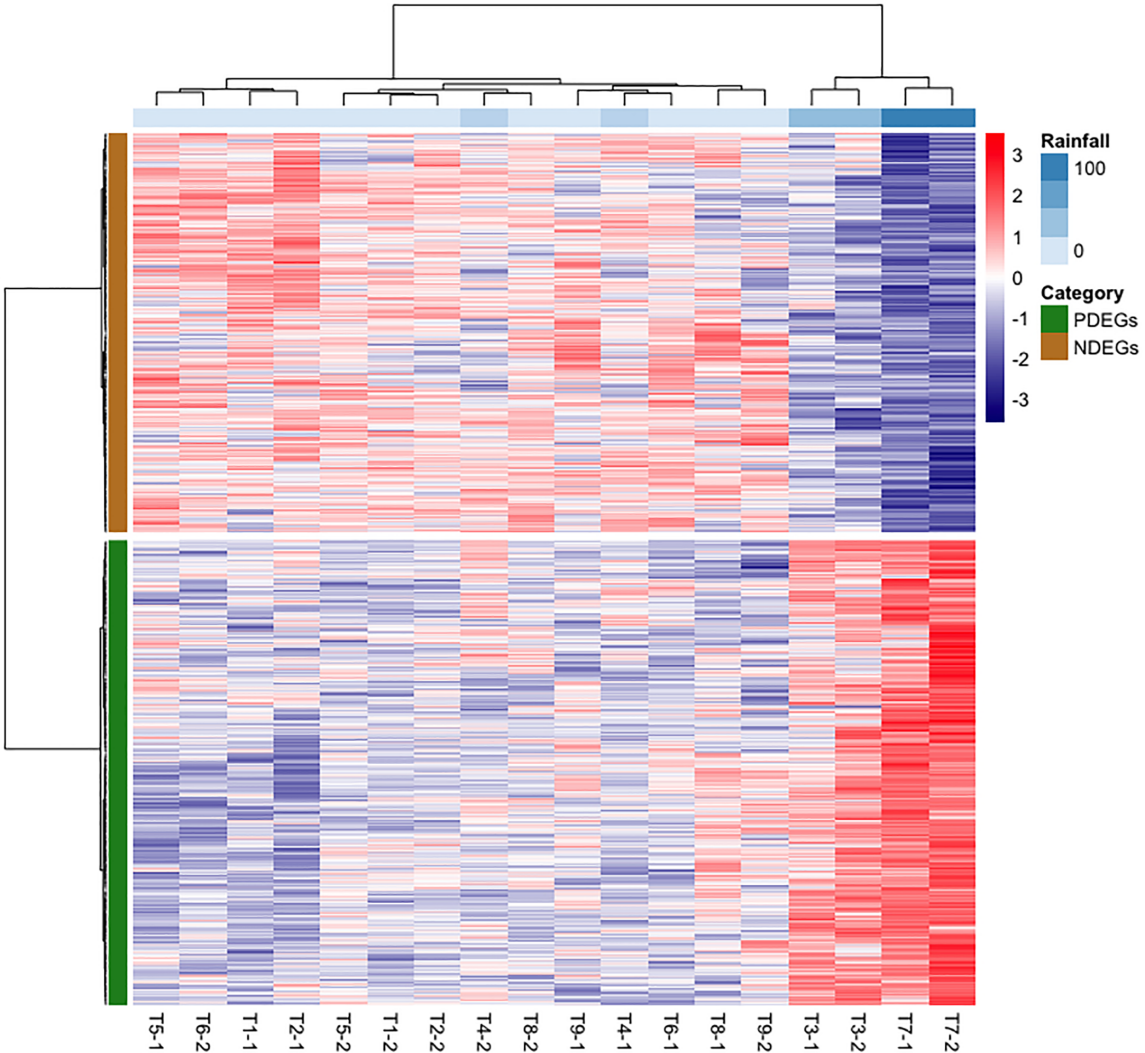
Stem core transcriptomic expression patterns of mature oil palms at the Lampung plantation correlated with cumulative precipitation over two-day periods. The red and purple colour scales represent the relative expression levels from higher to lower expression. PDEGs (green) and NDEGs (brown) are genes exhibiting positive and negative differential expression when correlated with cumulative precipitation over two days, respectively. The labels TX–1 and TX–2 denote the two samples collected at time point TX.

To determine how the stem core DEGs responded to rainfall, we clustered the samples based on their expression patterns and visualized the results using a heatmap (**Figure 3**). The analysis grouped the T3 and T7 samples into one cluster that was distinct from the other samples. Both samples were collected in February, during periods of heavy rain: the cumulative precipitation over two days was 40.2 mm for T3 and 105.0 mm for T7 (**Figure 1**).

We also had obtained DEGs correlating precipitation using a different approach. In this approach, gene expression data of T3 and T7 was grouped together which then compared to another group consists of gene expression data from other sampling time points. The generated heatmap under this approach (**Figure S5A**) also presented similar trend as the former approach, which further confirmed the reliability of our findings. Intriguingly, the similarity scores obtained by comparing to former approach in PDEGs and NDEGs are 87% and 66%, respectively (**Figures S5B, S5C**). Based on these results, we can exclude the error rate of obtaining DEGs due to small sample size.

### 3.3 Generation of gene sets through comparative transcriptome analysis

**Figure 4:**
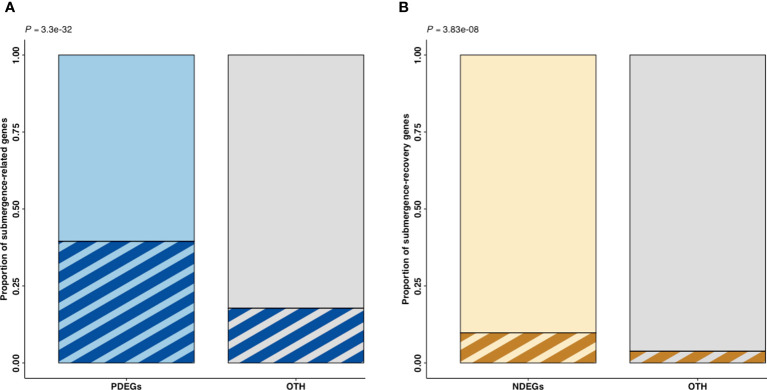
Proportion of submergence-related and submergence-recovery genes in the oil palm DEG sets identified by a comparative analysis. PDEGs (blue) and NDEGs (brown) are DEGs exhibiting positive and negative differential expression when correlated with cumulative precipitation over two days, respectively. The OTH represents gene sets that were excluded from either PDEGs or NDEGs. The p-value (P) indicates the significance of the difference between the oil palm DEG sets and **(A)** thaliana submergence DEG sets as determined using Fisher’s exact test. **(A)** PDEGs and OTH in the stem core **(B)** NDEGs and OTH in the stem core.

The PDEGs and NDEGs were overlapped with the DEGs identified in a submergence experiment using the model plant, *A. thaliana* (Yeung et al., 2018) to verify their involvement in waterlogging-induced stress responses or waterlogging recovery (**Figure 4**). In this analysis, submergence-related genes are defined as genes upregulated during submergence treatment in *A. thaliana* while submergence-recovery genes are genes upregulated during recovery from submergence.

Fisher’s exact test was used to evaluate the significance of the overlap between the oil palm DEGs and the *A. thaliana* submergence-related and submergence recovery genes. This revealed that the PDEG and NDEG gene sets contained significantly elevated proportions of submergence-related and submergence-recovery genes, respectively, when compared to a reference set of oil palm genes (**Figure 4A**). However, the proportion of *A. thaliana* submergence-recovery genes among the NDEGs was much lower that of submergence-related genes in the PDEGs.

### 3.4 GO and KEGG enrichment of PDEGs and NDEGs in oil palm core samples

**Figure 5:**
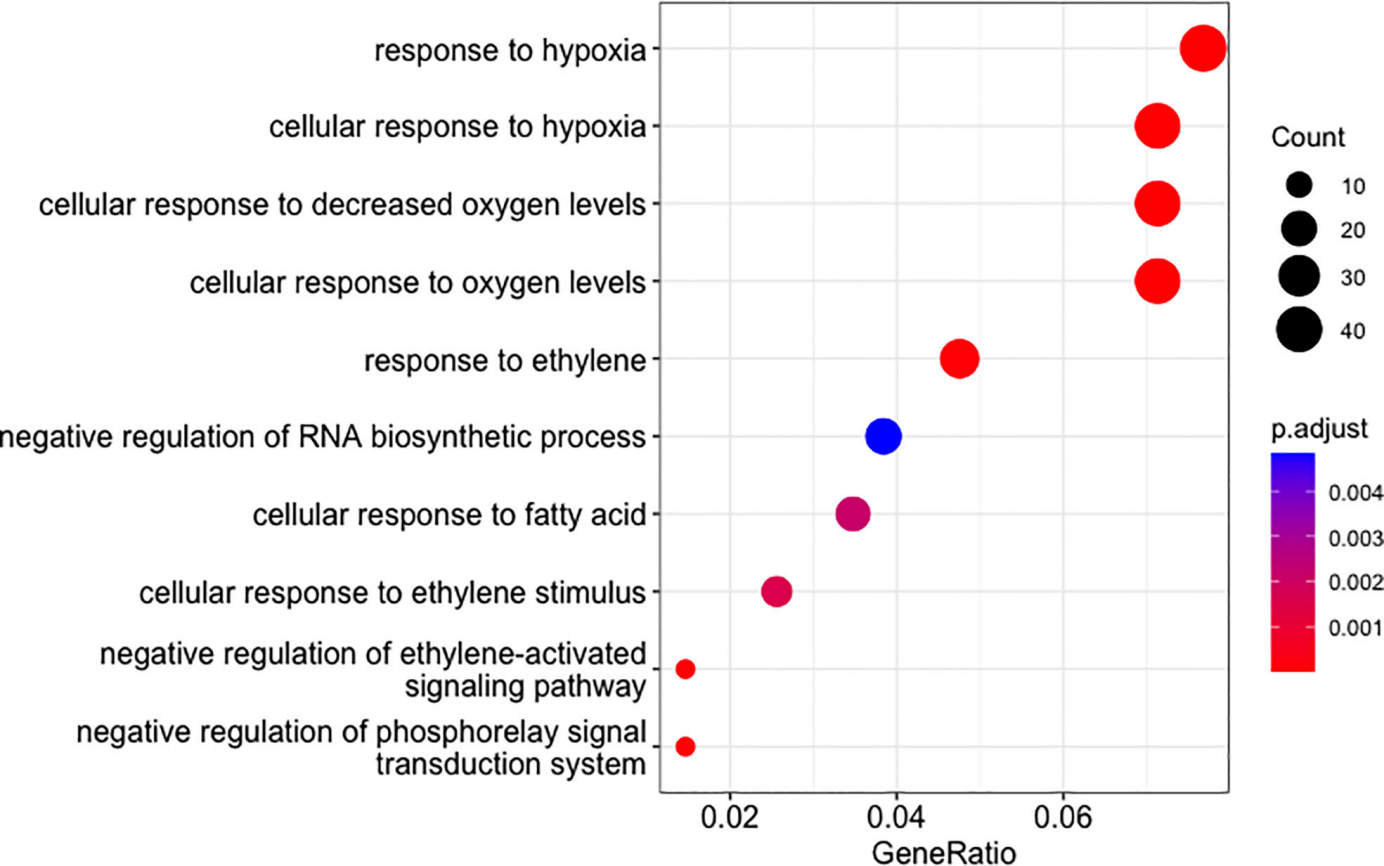
Enriched GO terms for DEGs whose expression correlated positively with cumulative precipitation over two days in oil palm stem core samples. The sizes of the dots indicate the enriched gene count and their colour indicates the p-values after adjustment using a Benjamini-Hochberg FDR of 0.05.

The results described above showed that the PDEGs and NDEGs identified in the oil palm stem core included significant numbers of waterlogging-related genes. For this reason, we only characterized the functions of these waterlogging-related PDEGs and NDEGs through GO and KEGG enrichment tests. The GO enrichment results of PDEGs revealed multiple GO terms that are closely related to waterlogging including response to hypoxia (GO:0071456, GO:0001666) and ethylene responses (GO:0009723, GO:0010105, GO:0071369) in PDEGs (**Figure 5** and **Supplementary Data 1**). The only GO term enriched among the NDEGs was response to heat (GO:0009408) (**Supplementary Data 1**).

We also performed a KEGG enrichment analysis with *A. thaliana* as the reference organism to identify the principal biological and signal transduction pathways of the waterlogging response. The PDEGs were significantly enriched in the glycolysis/gluconeogenesis pathway (ath00010), which is known to be generally stimulated in plants under hypoxic conditions. Additionally, the carotenoid biosynthesis pathway (ath00906) was enriched in the NDEGs (**Supplementary Data 1**).

### 3.5 Transcription factors regulating the expression of hypoxia-related genes

**Figure 6:**
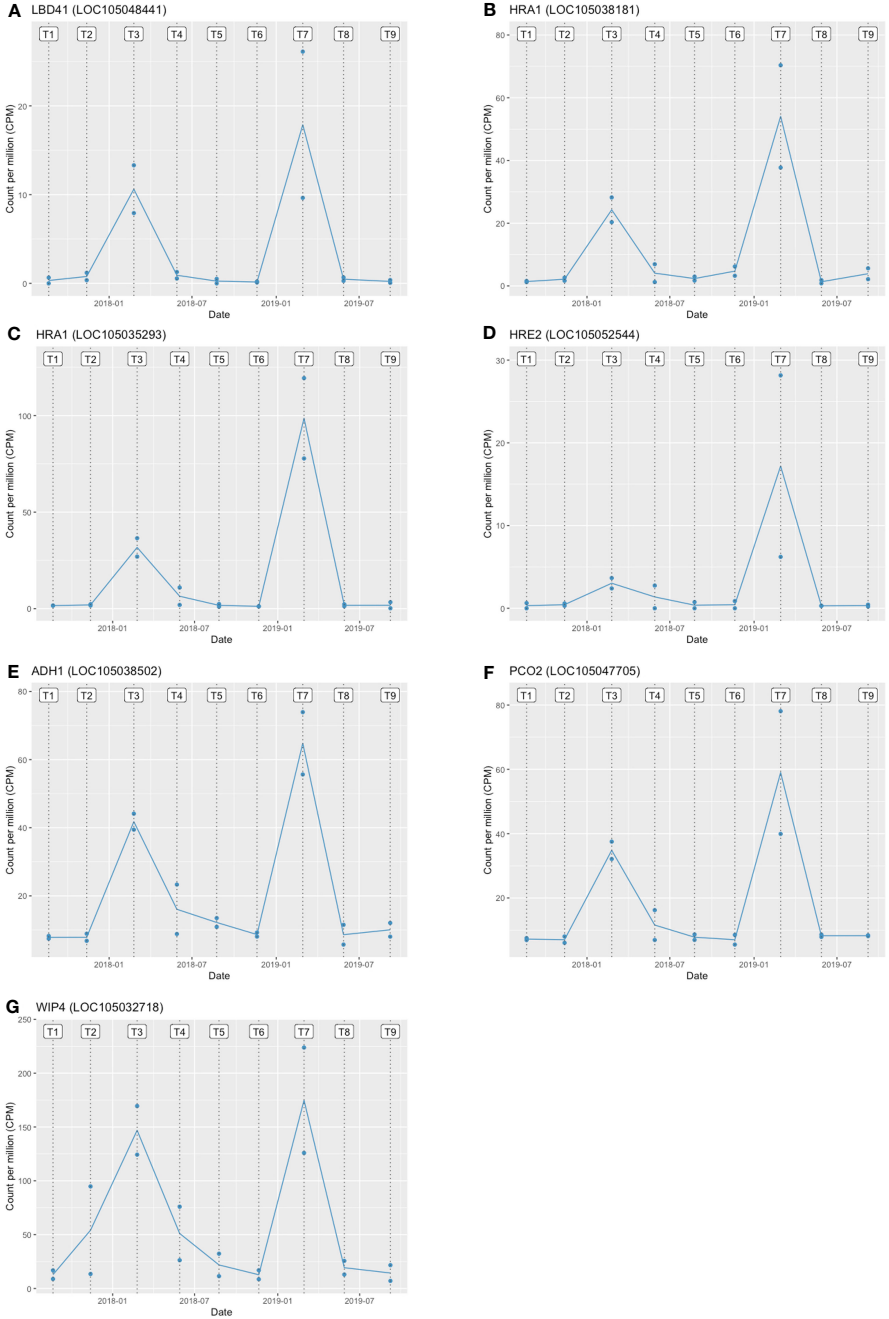
Expression patterns of hypoxia-related TFs and their target genes in oil palms during the experimental period. **(A)** *LBD41* **(B, C)** *HRA1* **(D)** *HRE2* are hypoxia-related TFs, and **(E)** *ADH1* **(F)** *PCO2* **(G)** *WIP4* are their target genes. T1–T9 denote the sampling time points. The two blue dots at each time point show the expression levels of the two stem core samples, and the line shows the mean expression level. Expression levels are plotted in units of counts per million (CPM).

The GO and KEGG enrichment analyses suggested the enrichment of hypoxia-related genes in the stem core DEGs. Thus, we further analysed the PDEGs, particularly response to hypoxia, which has been claimed as one of the critical repercussions of the waterlogging event in plants. To investigate this further, we performed a gene regulatory network (GRN) analysis of the PDEGs using GENIE3, which identified 19 candidate TFs associated with the PDEGs (**Table 1**). Among these are several TFs known to be involved in the hypoxia response of *A. thaliana*, namely *LBD41* (LOC105048441), *HRA1* (LOC105038181, LOC105035293), and *HRE2* (LOC105052544) (Eysholdt-Derzsó and Sauter, 2019). Consequently, we further examined the target genes of these four TFs and found some core hypoxia-related genes identified by Mustroph et al. (2009) in *A. thaliana* such as *ADH1* (LOC105038502), *PCO2* (LOC105047705) and *WIP4* (LOC105032718). Moreover, both of these candidate TFs as well as their gene targets were very strongly expressed at the T3 and T7 sampling points (**Figure 6**). The regulatory relationships of these TFs (**Tables 1**, **2**) and their seasonal expression patterns (**Figure 6**) strongly suggest that they and their gene targets are highly engaging with hypoxia and waterlogging responses in oil palm.

**Table 1:** Significant hypoxia-related TFs in the oil palm stem core.

Transcription factors	Descriptions	Adjusted *p-values*
LOC105041281	related to ABI3/VP1 2 (RAV2)	6.09E-12
LOC105051406	basic helix-loop-helix (bHLH) DNA-binding superfamily protein	1.12E-11
LOC105049728	auxin response factor 2 (ARF2)	1.03E-10
LOC105048441*	**LOB domain-containing protein 41 (LBD41)**	**2.72E-09**
LOC105055150	beta HLH protein 93 (bHLH093)	4.05E-09
LOC105038181*	**sequence-specific DNA binding transcription factors**	**9.29E-09**
LOC105038610	Plant regulator RWP-RK family protein	2.07E-07
LOC105052623	ALWAYS EARLY 3 (ALY3)	3.78E-07
LOC105035293*	**sequence-specific DNA binding transcription factors**	**1.93E-06**
LOC105058928	iaa-leucine resistant3 (ILR3)	1.25E-05
LOC105056257	cryptochrome-interacting basic-helix-loop-helix 1 (CIB1)	4.29E-05
LOC105041115	response regulator 2 (RR2)	2.46E-04
LOC105052544*	**HYPOXIA RESPONSIVE ERF (ETHYLENE RESPONSE FACTOR) 2 (HRE2)**	**3.58E-04**
LOC105047023	G-box binding factor 6 (GBF6)	6.15E-04
LOC105041457	Zinc finger C-x8-C-x5-C-x3-H type family protein	0.002078
LOC105057378	CHINESE FOR 'UGLY' (TSO1)	0.002652
LOC105038344	sequence-specific DNA binding transcription factors	0.013223
LOC105038777	SUPPRESSOR OF GAMMA RADIATION 1 (SOG1)	0.026485
LOC105059673	beta HLH protein 93 (bHLH093)	0.030111

*Expression patterns (bold) shown in **Figure 6**.

**Table 2:** Significant hypoxia-related genes identified in the oil palm stem core by GRN analysis.

Target genes	Descriptions	Transcription factors
LOC105032718*	**Wound-responsive family protein**	**LOC105048441**
LOC105034150	LOB domain-containing protein 41 (LBD41)	LOC105035293
LOC105034334	unknown protein	LOC105048441
LOC105038502*	**alcohol dehydrogenase 1 (ADH1)**	**LOC105035293**
LOC105047965	ACC oxidase 1 (ACO1)	LOC105052544
LOC105035293	sequence-specific DNA binding transcription factors	LOC105048441
LOC105047705*	**Protein of unknown function (DUF1637)**	**LOC105038181**
LOC105038181	sequence-specific DNA binding transcription factors	LOC105052544

*Expression patterns (bold) shown in **Figure 6**.

## 4 Discussion

### 4.1 Correlation between cumulative precipitation and waterlogging-related DEGs in oil palm

The number of detected DEGs was highest in samples collected during periods of heavy rainfall in February when aggregating precipitation over a time frame of 2 to 3 days. This is consistent with the findings of an earlier study on waterlogging in oil palms, which claimed that two days of waterlogging stress was detrimental to the secondary roots and three days of waterlogging stress led to elevated mRNA expression of genes responsible for lysigenous aerenchyma formation (Nuanlaong et al., 2021).

The rainy season of Lampung generally extends from December to February, so it is very likely that the studied oil palm plantation would have been waterlogged during this period. This likelihood is increased by the plantation’s flatland topography, which facilitates water stagnation and makes it highly prone to waterlogging following extreme rainfall (McFarlane, 1985; Valipour, 2014). Eventually, the soil profile of the area will become saturated with water, resulting in a perched water table in which the soil pores are filled with water instead of the air that plants need for normal respiratory activity. This can profoundly affect plant growth and development. A previous study stated that oil palms are quite tolerant of high-water tables but are nevertheless vulnerable to continuous waterlogging, which may provoke stress responses similar to those induced by drought (Rivera-Mendes et al., 2016). Some studies have projected waterlogging is common in Sumatra during the rainy season, and the Indonesian government has even listed Lampung as a city at severe risk due to climate anomalies (Supari et al., 2017; Supari et al., 2019; Tangang et al., 2020; Mukhlis and Perdana, 2022). Additionally, many studies have affirmed that oil palm requires an even distribution of annual rainfall with a total precipitation of at least 2000 mm per annum or 100 mm per month for optimal growth (Hartley, 1988; Corley and Tinker, 2003; Carr, 2011; Paramananthan, 2013). The cumulative precipitation over just two days was in excess of 30 mm at the T3 and T7 sampling points, we thus reasonably conclude that waterlogging occurred at both of these sampling points in the experimental oil palm field. Moreover, by scrutinizing the transcriptomic changes that occurred at the T3 and T7 time points, we identified DEGs involved in the transcriptomic waterlogging responses of adult oil palms. By reason of the number of identified DEGs was highest when aggregating precipitation over 2-day periods, we postulated that 2 days of waterlogging were needed to initiate the waterlogging response of oil palms correlating to the seasonal rainfall profile in the experimental plantation.

### 4.2 Waterlogging caused significant changes in gene expression in oil palm stem core

Previous studies on waterlogging in oil palms have examined seedlings cultivated under controlled conditions (Yulia and Sitorus, 2012; Rivera-Mendes et al., 2016; Cui et al., 2019; Nuanlaong et al., 2020; Nuanlaong et al., 2021). The goal of this study was to complement these works by studying waterlogging responses in adult palms growing under natural conditions, which is important because Poorter et al. (2016) have shown that differences between controlled and field environments can significantly affect plant performance and growth (Poorter et al., 2016; Forero et al., 2019). Additionally, previous studies on oil palm waterlogging have focused on the response in the roots, paying little attention to other organs. Although the roots exhibit a strong waterlogging response, responses in other organs also warrant study because systemic signals allow plants to respond to stress at the whole-plant level rather than merely at the local or cellular level (Zandalinas et al., 2020; Omary et al., 2023). Accordingly, there are numerous research publications have elucidated organ-specific waterlogging responses in other plants (Ellis et al., 1999; Mustroph et al., 2009; Hsu et al., 2011; Mustroph et al., 2014; Lothier et al., 2020; Cao et al., 2022) marked the necessity to investigate waterlogging responses in other parts of oil palm as well.

Herein, we studied the transcriptomic responses in stem core and leaves of oil palm towards waterlogging. This revealed that the samples collected at the T3 and T7 time points had very similar gene expression profiles that differed markedly from those seen at other time points. Moreover, an expression heatmap revealed significant transcriptomic changes in the oil palm stem core (**Figure 3**) whereas only minimal transcriptomic changes were detected in the leaves of the studied plants (**Figure 2** , **S2**). This was unexpected because the leaves of oil palm seedlings exhibited significant waterlogging-induced physiological and/or morphological changes in earlier studies (Yulia and Sitorus, 2012; Rivera-Mendes et al., 2016; Cui et al., 2019; Nuanlaong et al., 2020). The reliability of these findings was evaluated using Fisher’s exact test, which indicated that genes associated with waterlogging were significantly overrepresented among the DEGs identified in the stem core, while the opposite was true for the leaves (**Figure 4A, B**).

To date, both transcriptomic and physiological changes were observed in oil palm leaves as waterlogging responses reported by the previous studies, however, not found in adult oil palms according to our study. The discrepancies between the results could be arising by several factors. First, it could simply be the result of age-related differences in developmental stage (Gill, 1970; Glenz et al., 2006; Rankenberg et al., 2021): previous controlled experiments examined oil palm seedlings that were usually under one year old. It should be noted that oil palm seedlings are only transferred from nurseries to field environments when they are at least one year old; proper acclimatization and intensive cares are compulsory for their survivals (Turner and Gillbanks, 1974; Mutert et al., 1999; Corley and Tinker, 2015). Moreover, these oil palm seedlings are very sensitive to damage that may occur in natural environments due to harsh conditions, diseases, or pests (Corley and Tinker, 2015). The difference in responses may also be related to the fact that seedlings in developmental stage 1 have less well-developed systemic signalling capabilities than mature oil palms and lack a visible stem; many studies have shown that plant stems can play important roles in environmental stress responses (Gomes and Prado, 2007; Legros et al., 2009; Carr, 2011; Forero et al., 2012; da Ponte et al., 2019; Tani et al., 2020). The duration of the waterlogging event may also be important; this study focused mainly on transcriptomic changes detected after 2-3 days of heavy precipitation, which might not be long enough to observe waterlogging effects in oil palm leaves. Longer periods of waterlogging were imposed in some earlier controlled studies where such responses were seen (Rivera-Mendes et al., 2016; Cui et al., 2019). Finally, mature oil palms can have stem heights of 20 – 30 m and are thus much bigger than seedlings (Corley and Tinker, 2015). Consequently, the leaves of adult oil palms are much further from the ground than those of seedlings and may be more sensitive to changes in the aboveground environment than those below ground. The absence of detectable waterlogging responses in oil palm leaves is consistent with the results of an earlier waterlogging study on poplar trees (Kreuzwieser et al., 2009). These factors may explain why our results are largely consistent with previously reported waterlogging responses but exhibit some disparities at the organ-specific level.

Although this is the first published transcriptomic analysis of waterlogging responses in oil palm stems, the validity of the results presented here is quite justifiable from several aspects. First, a significant waterlogging response was detected in the stem core even though the stem core samples were collected 1 m below the branch of the lowest frond, which is close to the leaves and very far from the roots. This is important because the roots are generally considered to be the organ most strongly involved in detecting and responding to waterlogging, our results show that waterlogging can induce organ-specific changes in expression elsewhere in the plant. The oil palm stem has been notable for functioning as non-structural carbohydrate depository that serves as a buffer to compensate for seasonal source-sink imbalances, allowing the plant to grow and survive under adverse environmental conditions (Legros et al., 2009; Tani et al., 2020). Its potential involvement in waterlogging responses is supported by the fact that some flood-tolerant plant species display morphological or anatomical adaptations such as hypertrophied lenticels at the stem base that allow gas exchange to occur in the stem in the event of waterlogging (Glenz et al., 2006; Parelle et al., 2007; Shimamura et al., 2010; Rivera-Mendes et al., 2016). Many publications have also concluded that stem flows in trees vary depending on growth stage and size, the species’ waterlogging tolerance, and the duration and intensity of the waterlogging event (Glenz et al., 2006; Carr, 2011). Finally, it should be noted that the soil water content depends on the soil type and geographical factors, and that uncontrolled variation in these parameters could potentially overpower the effects of changes in precipitation when analysing the effects of waterlogging and the resulting plant responses.

### 4.3 Hypoxia and ethylene signalling as the main waterlogging responses in oil palm

Waterlogging causes hypoxia (i.e., oxygen deficiency) in the roots of waterlogged plants (Parent et al., 2008; Loreti and Perata, 2020) by virtue of difficulties in gaseous exchange. Accordingly, the most significantly enriched GO terms among the palm oil stem PDEGs were related to hypoxia and reduced oxygen levels (GO:0071456, GO:0036294, GO:0071453, GO:0001666). Terms associated with ethylene responses (GO:0009723, GO:0010105, GO:0071369) were also heavily enriched. Ethylene is a prominent phytohormone that is involved in almost every biological process in plants, particularly those associated with stress responses, development, and growth (Johnson and Ecker, 1998; Ali and Kim, 2018). It is also crucial for stimulating the development of morphological-anatomical features that facilitate adaptation to or tolerance of waterlogging such as hypertrophied lenticels, adventitious roots, and aerenchyma (Ali and Kim, 2018). Several genes associated with ethylene were highly expressed in waterlogged roots of Deli x Ghana (GSR) oil palms, including *ACS3*, *ACO*, and *ACO1* (Nuanlaong et al., 2020). It is therefore notable that *ACO1* (LOC105047965) was one of the PDEGs identified after heavy rainfall event in the plantation. ACC oxidase (*ACO*) is known to involve in the conversion of 1-aminocyclopropane 1-carboxylic acid (ACC) into ethylene and in biological processes such as hormonal crosstalk and abiotic stress responses (Pattyn et al., 2021).

The remaining GO terms enriched among the PDEGs also appear to be related to waterlogging responses because they are associated with environmental stress responses including defence, immune, and stimuli responses as well as aging, nutrient homeostasis, and plant growth (**Figure 5** and **Supplementary Data 1**). One of the KEGG terms enriched in the PDEGs was the glycolysis/gluconeogenesis pathway (ath00010), which was expected because this pathway is known to be ubiquitous in plant waterlogging responses, compensating for oxygen and energy deficiencies via fermentation processes or anaerobic respiration. The carbon metabolism (ath01200) and carbon fixation (ath00710) pathways, which are both related to sugar metabolism, were also significantly enriched. This suggests that waterlogging induces increased starch and sucrose degradation to maintain a constant carbohydrate supply to waterlogged plants (Parent et al., 2008; Mustroph et al., 2009; Kreuzwieser and Rennenberg, 2014; Planchet et al., 2017; Cui et al., 2019; Lothier et al., 2020). Similar findings were obtained in an earlier transcriptomic study on oil palm seedlings, supporting our results (Nuanlaong et al., 2020).

Waterlogging has been characterized as a sequential stress like drought stress, meaning that there are distinct stress responses during and after the waterlogging occurrence (Striker, 2012; Yeung et al., 2018; Wang et al., 2021). The post-stress recovery response prepares the plant for growth resumption and is therefore important for overall stress tolerance assessment of the plant. When water recedes, the abrupt exposure of waterlogged plant parts to normoxic conditions can induce abiotic stress. Any mechanisms or processes initiated during this period could be regarded as elements of the post-waterlogging recovery response. Thus far, no study has been explored on the recovery of waterlogged oil palm as oil palm was previously thought to be waterlog- or flood-tolerant until recent. Myriad of stresses observed in *A. thaliana* during post-waterlogging recovery are predominantly oxidative, osmotic, light, and temperature (heat) stresses (Yeung et al., 2018). This is consistent with the finding that the only GO and KEGG terms enriched in the oil palm stem NDEG set, which consists of candidate waterlogging-recovery genes, were response to heat (GO:0009408) and the carotenoid biosynthesis pathway (ath00906).

Waterlogging events in tropical regions are often accompanied by reductions in temperature that could alleviate heat stress during hot weather or exacerbate cold stress during cold periods. Plants frequently suffer heat stress due to upsurge in environmental temperatures after water subsides, especially in tropical countries like Indonesia and Malaysia. Considering climates in these countries, temperature stress as in heat stress is more likely to be imposed on oil palms after stagnant soil water went off. Moreover, the carotenoid biosynthesis pathway, which is important in plant hormone signal transduction during drought responses, was found to be significantly enriched in a study on drought tolerance in oil palms (Wang et al., 2020). These results therefore suggest that oil palms in plantations are exposed to transient drought-mimicking conditions after waterlogging and the sudden drainage of soil water. Aside from this, carotenoids are known eminently for involving in photosynthesis, counteractive mechanism against abiotic stresses, phytohormone synthesis, and stress tolerance improvement (Mi et al., 2022; Pais et al., 2023). Apart from temperature stress, other stresses that usually arise post-waterlogging recovery period are infinitesimal. As the case may be the tall figure of adult oil palm, the waterlogging depth could be shallow and waterlogging effect is unreachable to leaves that are often exposed to illumination stress.

### 4.4 ERF-VII transcription factors are involved in hypoxia and waterlogging responses in oil palms

The transcriptomic response to waterlogging in adult oil palms is less well characterized than that in the model plant *A. thaliana*, so a comparative analysis with *A. thaliana* was performed to identify the transcription factors involved in the waterlogging response in adult oil palms (Ma et al., 2005; Kobayashi et al., 2013). A GRN analysis revealed a total of 19 relevant TFs, four of which are known to be involved in responses to hypoxia. The association between the known functions of the remaining 15 TFs and the responses to hypoxia and waterlogging in *A. thaliana* are unclear.

One of the four hypoxia-related TFs identified in the GRN was *HRE2*, which belongs to the ERF-VII TF family. ERF-VII TFs play vital roles in responses to waterlogging and hypoxia in *A. thaliana* because they influence oxygen sensing via the N-end rule pathway, which regulates proteasomal degradation under oxygen-rich condition and vice versa under hypoxic condition. The N-end rule pathway is a highly conserved protein stabilization system in both plants and mammals (Licausi et al., 2011; Eysholdt-Derzsó and Sauter, 2019). A conserved N-terminal motif (MCGGAI/L, termed the MC motif) in ERF-VIIs plays an essential role in this degradation pathway and is present in the HRE2 protein (LOC105052544), supporting the involvement of *HRE2* in the response to waterlogging-induced hypoxia in oil palms (**Figure S6**).

Another TF identified in the GRN analysis was bHLH, whose functions in the oil palm waterlogging response are unclear. However, the bHLH family is one of the largest TF families in *A. thaliana* and has pleiotropic regulatory effects. Additionally, bHLH TF possessed a highly conserved motif that could be found universally in eukaryotic organisms (Hao et al., 2021). It is also notable that some of the TFs identified in the GRN analysis are involved in ethylene responses, namely *RAV2* (LOC105041281) and *ARF2* (LOC105049728). This is notable because ethylene signalling is essential in waterlogging responses (Li et al., 2006; Wang et al., 2011; Fu et al., 2014; Zhou and Yarra, 2021). Future studies to elucidate the functions of these TFs and their respective gene targets in waterlogged oil palms could deepen our understanding of waterlogging mechanisms in oil palm.

## 5 Conclusions

Waterlogging responses in oil palms have previously been studied using seedlings maintained under controlled conditions. While such studies can provide valuable insights, their results are not always directly applicable to mature plants grown in field environments. Moreover, it is impossible to perfectly replicate field conditions in controlled environments because sessile plants are naturally exposed to multifactorial stress combinations. The results presented here thus complement those of earlier studies. Organ-specific transcriptional responses to waterlogging were detected in mature oil palms growing in the field, with significant transcriptional changes occurring in stem core tissue but not in leaves. This is inconsistent with findings from controlled studies on waterlogged oil palm seedlings. However, the main waterlogging responses observed in adult oil palm stems were consistent with previously reported plant waterlogging responses involving adaptation to hypoxia and ethylene signalling. Several candidate TFs and gene targets involved in the waterlogging response were also identified, with the *HRE2* TF belonging to the ERF-VII family being postulated to have a particularly important role. Further studies on the age-dependence of the waterlogging response and the mechanistic roles of the identified TFs and their target genes will be needed to obtain a comprehensive understanding of the waterlogging response in oil palms.

## Data availability statement

All read files generated for this study can be found in NCBI SRA database under BioProject accession number PRJNA961916 (https://www.ncbi.nlm.nih.gov/bioproject/PRJNA961916).

## Author contributions

NT and AK originated research idea; NT, SM, DI and TK designed the study; NT, SM and DI collected samples; HL, SM and DI collected meteorological data; HL, SM, DI and NT processed samples and RNA extraction; HL and MK analyzed RNA-seq data; HL drafted manuscript; HL, MK and NT edited the manuscript. All the authors contributed to checking and finalization of the manuscript. 

## References

[B1] Abubakar A. Ishak M. Y. Makmom A. A. (2021). Impacts of and adaptation to climate change on the oil palm in Malaysia: a systematic review. Environ. Sci. pollut. Res. Int. 28, 54339–54361. doi: 10.1007/s11356-021-15890-3 34402002 PMC8494663

[B2] Ali S. Kim W.-C. (2018). Plant growth promotion under water: decrease of waterlogging-induced ACC and ethylene levels by ACC deaminase-producing bacteria. Front. Microbiol. 9. doi: 10.3389/fmicb.2018.01096 PMC5981179 29887854

[B3] Altschul S. F. Gish W. Miller W. Myers E. W. Lipman D. J. (1990). Basic local alignment search tool. J. Mol. Biol. 215, 403–410. doi: 10.1016/s0022-2836(05)80360-2 2231712

[B4] Ashburner M. Ball C. A. Blake J. A. Botstein D. Butler H. Cherry J. M.. (2000). Gene Ontology: tool for the unification of biology. Nat. Genet. 25, 25–29. doi: 10.1038/75556 10802651 PMC3037419

[B5] Benjamini Y. Hochberg Y. (1995). Controlling the false discovery rate: a practical and powerful approach to multiple testing. J. R. Stat. Soc Ser. B 57, 289–300. doi: 10.1111/j.2517-6161.1995.tb02031.x

[B6] Bolger A. M. Lohse M. Usadel B. (2014). Trimmomatic: a flexible trimmer for Illumina sequence data. Bioinformatics 30, 2114–2120. doi: 10.1093/bioinformatics/btu170 24695404 PMC4103590

[B7] Cao M. Zheng L. Li J. Mao Y. Zhang R. Niu X.. (2022). Transcriptomic profiling suggests candidate molecular responses to waterlogging in cassava. PLoS One 17, e0261086. doi: 10.1371/journal.pone.0261086 35061680 PMC8782352

[B8] Carr M. K. V. (2011). The water relations and irrigation requirements of oil palm (*Elaeis guineensis*): a review. Exp. Agric. 47, 629–652. doi: 10.1017/S0014479711000494

[B9] Corley R. H. V. Tinker P. B. (2003). The oil palm. 4th ed (Hoboken, New Jersey, United States: Oxford: Blackwell Science). doi: 10.1002/9780470750971

[B10] Corley R. H. V. Tinker P. B. (2015). The oil palm. 5th ed (Hoboken, New Jersey, United States: Wiley-Blackwell).

[B11] Cui J. Davanture M. Zivy M. Lamade E. Tcherkez G. (2019). Metabolic responses to potassium availability and waterlogging reshape respiration and carbon use efficiency in oil palm. New Phytol. 223, 310–322. doi: 10.1111/nph.15751 30767245

[B12] da Ponte N. H. T. Nunes Santos R. I. Lima Lopes Filho W. R. Lisboa Cunha R. Murad Magalhães M. Alves Pinheiro H. (2019). Morphological assessments evidence that higher number of pneumatophores improves tolerance to long-term waterlogging in oil palm (*Elaeis guineensis*) seedlings. Flora 250, 52–58. doi: 10.1016/j.flora.2018.11.017

[B13] Ellis M. H. Dennis E. S. James Peacock W. (1999). Arabidopsis roots and shoots have different mechanisms for hypoxic stress tolerance. Plant Physiol. 119, 57–64. doi: 10.1104/pp.119.1.57 9880346 PMC32242

[B14] Eysholdt-Derzsó E. Sauter M. (2019). Hypoxia and the group VII ethylene response transcription factor HRE2 promote adventitious root elongation in *Arabidopsis* . Plant Biol. 21, 103–108. doi: 10.1111/plb.12873 29996004 PMC6585952

[B15] Forero L. E. Grenzer J. Heinze J. Schittko C. Kulmatiski A. (2019). Greenhouse- and field-measured plant-soil feedbacks are not correlated. Front. Environ. Sci. 7. doi: 10.3389/fenvs.2019.00184

[B16] Forero D. C. Hormaza P. Romero H. M. (2012). Phenological growth stages of African oil palm (*Elaeis guineensis*). Ann. Appl. Biol. 160, 56–65. doi: 10.1111/j.1744-7348.2011.00520.x

[B17] Fu M. Kang H. K. Son S.-H. Kim S.-K. Nam K. H. (2014). A subset of Arabidopsis RAV transcription factors modulates drought and salt stress responses independent of ABA. Plant Cell Physiol. 55, 1892–1904. doi: 10.1093/pcp/pcu118 25189341

[B18] Gill C. J. (1970). The flooding tolerance of woody species - a review. Forestry Abstract 31, 671–688.

[B19] Glenz C. Schlaepfer R. Iorgulescu I. Kienast F. (2006). Flooding tolerance of Central European tree and shrub species. For Ecol. Manage 235, 1–13. doi: 10.1016/j.foreco.2006.05.065

[B20] Gomes F. P. Prado C. H. B. A. (2007). Ecophysiology of coconut palm under water stress. Braz. J. Plant Physiol. 19, 377–391. doi: 10.1590/S1677-04202007000400008

[B21] Hao Y. Zong X. Ren P. Qian Y. Fu A. (2021). Basic helix-loop-helix (bHLH) transcription factors regulate a wide range of functions in *Arabidopsis* . Int. J. Mol. Sci. 22, 7152. doi: 10.3390/ijms22137152 34281206 PMC8267941

[B22] Hartley C. W. S. (1988). The oil palm. 3rd ed (London: Longman).

[B23] Hsu F.-C. Chou M.-Y. Peng H.-P. Chou S.-J. Shih M.-C. (2011). Insights into hypoxic systemic responses based on analyses of transcriptional regulation in Arabidopsis. PloS One 6, e28888. doi: 10.1371/journal.pone.0028888 22194941 PMC3240646

[B24] Johnson P. R. Ecker J. R. (1998). The ethylene gas signal transduction pathway: a molecular perspective. Annu. Rev. Genet. 32, 227—254. doi: 10.1146/annurev.genet.32.1.227 9928480

[B25] Kanehisa M. Goto S. (2000). KEGG: kyoto encyclopedia of genes and genomes. Nucleic Acids Res. 28, 27–30. doi: 10.1093/nar/28.1.27 10592173 PMC102409

[B26] Kobayashi M. J. Takeuchi Y. Kenta T. Kume T. Diway B. Shimizu K. K. (2013). Mass flowering of the tropical tree *Shorea beccariana* was preceded by expression changes in flowering and drought-responsive genes. Mol. Ecol. 22, 4767–4782. doi: 10.1111/mec.12344 23651119 PMC3817532

[B27] Kreuzwieser J. Hauberg J. Howell K. A. Carroll A. Rennenberg H. Millar A. H.. (2009). Differential response of gray poplar leaves and roots underpins stress adaptation during hypoxia. Plant Physiol. 149, 461–473. doi: 10.1104/pp.108.125989 19005089 PMC2613732

[B28] Kreuzwieser J. Rennenberg H. (2014). Molecular and physiological responses of trees to waterlogging stress. Plant Cell Environ. 37, 2245–2259. doi: 10.1111/pce.12310 24611781

[B29] Langan P. Bernád V. Walsh J. Henchy J. Khodaeiaminjan M. Mangina E.. (2022). Phenotyping for waterlogging tolerance in crops: current trends and future prospects. J. Exp. Bot. 73, 5149–5169. doi: 10.1093/jxb/erac243 35642593 PMC9440438

[B30] Legros S. Mialet-Serra I. Clement-Vidal A. Caliman J.-P. Siregar F. A. Fabre D.. (2009). Role of transitory carbon reserves during adjustment to climate variability and source–sink imbalances in oil palm (*Elaeis guineensis*). Tree Physiol. 29, 1199–1211. doi: 10.1093/treephys/tpp057 19675073

[B31] Li J. Dai X. Zhao Y. (2006). A role for auxin response factor 19 in auxin and ethylene signaling in Arabidopsis. Plant Physiol. 140, 899–908. doi: 10.1104/pp.105.070987 16461383 PMC1400570

[B32] Licausi F. Kosmacz M. Weits D. A. Giuntoli B. Giorgi F. M. Voesenek L. A. C. J.. (2011). Oxygen sensing in plants is mediated by an N-end rule pathway for protein destabilization. Nature 479, 419–422. doi: 10.1038/nature10536 22020282

[B33] Loreti E. Perata P. (2020). The many facets of hypoxia in plants. Plants 9, 745. doi: 10.3390/plants9060745 32545707 PMC7356549

[B34] Lothier J. Diab H. Cukier C. Limami A. M. Tcherkez G. (2020). ). Metabolic responses to waterlogging differ between roots and shoots and reflect phloem transport alteration in *Medicago truncatula* . Plants 9, 1373. doi: 10.3390/plants9101373 33076529 PMC7650564

[B35] Ma L. Chen C. Liu X. Jiao Y. Su N. Li L.. (2005). A microarray analysis of the rice transcriptome and its comparison to Arabidopsis. Genome Res. 15, 1274–1283. doi: 10.1101/gr.3657405 16140994 PMC1199542

[B36] McFarlane D. J. (1985). Assessment of waterlogged sites. J. Department Agriculture Western Australia Ser. 4 26, 119–121.

[B37] Mi J. Vallarino J. G. Petřík I. Novák O. Correa S. M. Chodasiewicz M.. (2022). A manipulation of carotenoid metabolism influence biomass partitioning and fitness in tomato. Metab. Eng. 70, 166–180. doi: 10.1016/j.ymben.2022.01.004 35031492

[B38] Miyazawa Y. Tateishi M. Komatsu H. Ma V. Kajisa T. Sokh H.. (2014). Tropical tree water use under seasonal waterlogging and drought in central Cambodia. J. Hydrol (Amst) 515, 81–89. doi: 10.1016/j.jhydrol.2014.04.049

[B39] Mukhlis M. Perdana R. (2022). A critical analysis of the challenges of collaborative governance in climate change adaptation policies in Bandar Lampung city, Indonesia. Sustainability 14, 4077. doi: 10.3390/su14074077

[B40] Mustroph A. Barding JR G. A. Kaiser K. A. Larive C. K. Bailey-Serres J. (2014). Characterization of distinct root and shoot responses to low-oxygen stress in Arabidopsis with a focus on primary C- and N-metabolism. Plant Cell Environ. 37, 2366–2380. doi: 10.1111/pce.12282 24450922

[B41] Mustroph A. Zanetti M. E. Jang C. J. H. Holtan H. E. Repetti P. P. Galbraith D. W.. (2009). Profiling translatomes of discrete cell populations resolves altered cellular priorities during hypoxia in *Arabidopsis* . Proc. Natl. Acad. Sci. 106, 18843–18848. doi: 10.1073/pnas.0906131106 19843695 PMC2764735

[B42] Mutert E. Esquìvez A. S. de los Santos A. O. Cervantes E. O. (1999). The oil palm nursery: foundation for high production. Better Crops Int. 13, 39–44.

[B43] Nuanlaong S. Wuthisuthimethavee S. Mekanawakul M. Suraninpong P. (2020). Transcriptome analysis of oil palm (*Elaeis guineensis Jacq.*) roots under waterlogging stress. Plant Omics 13, 46–56. doi: 10.21475/POJ.13.01.20.p2327

[B44] Nuanlaong S. Wuthisuthimethavee S. Suraninpong P. (2021). Lysigenous aerenchyma formation: responsiveness to waterlogging in oil palm roots. Biol. Plant 65, 167–176. doi: 10.32615/bp.2021.002

[B45] Omary M. Matosevich R. Efroni I. (2023). Systemic control of plant regeneration and wound repair. New Phytol. 237, 408–413. doi: 10.1111/nph.18487 36101501 PMC10092612

[B46] Pais I. P. Moreira R. Semedo J. N. Ramalho J. C. Lidon F. C. Coutinho J.. (2023). Wheat crop under waterlogging: potential soil and plant effects. Plants 12, 149. doi: 10.3390/plants12010149 PMC9823972 36616278

[B47] Paramananthan S. (2013). Managing marginal soils for sustainable growth of oil palms in the tropics. J. Oil Palm Environ. 4, 1–16. doi: 10.5366/jope.2013.1

[B48] Parelle J. Brendel O. Jolivet Y. Dreyer E. (2007). Intra- and interspecific diversity in the response to waterlogging of two co-occurring white oak species (*Quercus robur and Q. petraea*). Tree Physiol. 27, 1027–1034. doi: 10.1093/treephys/27.7.1027 17403656

[B49] Parent C. Capelli N. Berger A. Crèvecoeur M. Dat J. F. (2008). An overview of plant responses to soil waterlogging. Plant Stress 2, 20–27.

[B50] Paterson R. R. M. Lima N. (2018). Climate change affecting oil palm agronomy, and oil palm cultivation increasing climate change, require amelioration. Ecol. Evol. 8, 452–461. doi: 10.1002/ece3.3610 29321885 PMC5756879

[B51] Paterson R. R. M. Sariah M. Lima N. (2013). How will climate change affect oil palm fungal diseases? Crop Prot. 46, 113–120. doi: 10.1016/j.cropro.2012.12.023

[B52] Pattyn J. Vaughan-Hirsch J. Van de Poel B. (2021). The regulation of ethylene biosynthesis: a complex multilevel control circuitry. New Phytol. 229, 770–782. doi: 10.1111/nph.16873 32790878 PMC7820975

[B53] Pérez-Jiménez M. Pérez-Tornero O. (2021). Short-term waterlogging in citrus rootstocks. Plants 10, 2772. doi: 10.3390/plants10122772 34961243 PMC8704903

[B54] Pertea M. Kim D. Pertea G. M. Leek J. T. Salzberg S. L. (2016). Transcript-level expression analysis of RNA-seq experiments with HISAT, StringTie and Ballgown. Nat. Protoc. 11, 1650–1667. doi: 10.1038/nprot.2016.095 27560171 PMC5032908

[B55] Planchet E. Lothier J. Limami A. M. (2017). “Hypoxic respiratory metabolism in plants: reorchestration of nitrogen and carbon metabolisms,” in Plant respiration: metabolic fluxes and carbon balance. Eds. Tcherkez G. Ghashghaie J. (Cham: Springer International Publishing), 209–226. doi: 10.1007/978-3-319-68703-2_10

[B56] Poorter H. Fiorani F. Pieruschka R. Wojciechowski T. van der Putten W. H. Kleyer M.. (2016). Pampered inside, pestered outside? Differences and similarities between plants growing in controlled conditions and in the field. New Phytol. 212, 838–855. doi: 10.1111/nph.14243 27783423

[B57] Rankenberg T. Geldhof B. van Veen H. Holsteens K. Van de Poel B. Sasidharan R. (2021). Age-dependent abiotic stress resilience in plants. Trends Plant Sci. 26, 692–705. doi: 10.1016/j.tplants.2020.12.016 33509699

[B58] Rivera-Mendes Y. D. Cuenca J. C. Romero H. M. (2016). Physiological responses of oil palm (*Elaeis guineensis Jacq.*) seedlings under different water soil conditions. Agron. Colomb 34, 163–171. doi: 10.15446/agron.colomb.v34n2.55568

[B59] Robinson M. D. McCarthy D. J. Smyth G. K. (2010). edgeR: a Bioconductor package for differential expression analysis of digital gene expression data. Bioinformatics 26, 139–140. doi: 10.1093/bioinformatics/btp616 19910308 PMC2796818

[B60] Shimamura S. Yamamoto R. Nakamura T. Shimada S. Komatsu S. (2010). Stem hypertrophic lenticels and secondary aerenchyma enable oxygen transport to roots of soybean in flooded soil. Ann. Bot. 106, 277–284. doi: 10.1093/aob/mcq123 20660468 PMC2908175

[B61] Singh R. Ong-Abdullah M. Low E.-T. L. Manaf M. A. A. Rosli R. Nookiah R.. (2013). Oil palm genome sequence reveals divergence of interfertile species in Old and New worlds. Nature 500, 335–339. doi: 10.1038/nature12309 23883927 PMC3929164

[B62] Striker G. G. (2012). Time is on our side: the importance of considering a recovery period when assessing flooding tolerance in plants. Ecol. Res. 27, 983–987. doi: 10.1007/s11284-012-0978-9

[B63] Supari S. Linarka U. A. Rizal J. Satyaningsih R. Chung J. X. (2019). Indonesian climate under 2°C and 4°C global warming: precipitation extremes. IOP Conf Ser. Earth Environ. Sci. 303, 12048. doi: 10.1088/1755-1315/303/1/012048

[B64] Supari F. Juneng L. Aldrian E. (2017). Observed changes in extreme temperature and precipitation over Indonesia. Int. J. Climatol. 37, 1979–1997. doi: 10.1002/joc.4829

[B65] Swarbreck D. Wilks C. Lamesch P. Berardini T. Z. Garcia-Hernandez M. Foerster H.. (2008). The Arabidopsis Information Resource (TAIR): gene structure and function annotation. Nucleic Acids Res. 36, D1009–D1014. doi: 10.1093/nar/gkm965 17986450 PMC2238962

[B66] Takahashi F. Kuromori T. Urano K. Yamaguchi-Shinozaki K. Shinozaki K. (2020). Drought stress responses and resistance in plants: from cellular responses to long-distance intercellular communication. Front. Plant Sci. 11. doi: 10.3389/fpls.2020.556972 PMC7511591 33013974

[B67] Tangang F. Chung J. X. Juneng L. Supari, Salimun E. Ngai S. T.. (2020). Projected future changes in rainfall in Southeast Asia based on CORDEX–SEA multi-model simulations. Clim Dyn 55, 1247–1267. doi: 10.1007/s00382-020-05322-2

[B68] Tani N. Abdul Hamid Z. A. Joseph N. Sulaiman O. Hashim R. Arai T.. (2020). Small temperature variations are a key regulator of reproductive growth and assimilate storage in oil palm (*Elaeis guineensis*). Sci. Rep. 10, 650. doi: 10.1038/s41598-019-57170-8 31959766 PMC6971258

[B69] Tian L. Zhang Y. Chen P. Zhang F. Li J. Yan F. (2021)How does the waterlogging regime affect crop yield? A global meta-analysis Front. Plant Sci. 12, 634898. doi: 10.3389/fpls.2021.634898 33679848 PMC7933672

[B70] Torres G. A. Sarria G. A. Martinez G. Varon F. Drenth A. Guest D. I. (2016). Bud rot caused by *Phytophthora palmivora:* a destructive emerging disease of oil palm. Phytopathology 106, 320–329. doi: 10.1094/PHYTO-09-15-0243-RVW 26714102

[B71] Turner P. D. Gillbanks R. A. (1974). Oil palm cultivation and management (Kuala Lumpur: Incorporated Society of Planters).

[B72] Valipour M. (2014). Drainage, waterlogging, and salinity. Arch. Agron. Soil Sci. 60, 1625–1640. doi: 10.1080/03650340.2014.905676

[B73] Wang L. Dossa K. You J. Zhang Y. Li D. Zhou R.. (2021). High-resolution temporal transcriptome sequencing unravels ERF and WRKY as the master players in the regulatory networks underlying sesame responses to waterlogging and recovery. Genomics 113, 276–290. doi: 10.1016/j.ygeno.2020.11.022 33249174

[B74] Wang L. Hua D. He J. Duan Y. Chen Z. Hong X.. (2011). Auxin Response Factor2 (ARF2) and its regulated homeodomain gene HB33 mediate abscisic acid response in *Arabidopsis* . PloS Genet. 7, e1002172. doi: 10.1371/journal.pgen.1002172 21779177 PMC3136439

[B75] Wang L. Lee M. Ye B. Yue G. H. (2020). Genes, pathways and networks responding to drought stress in oil palm roots. Sci. Rep. 10, 21303. doi: 10.1038/s41598-020-78297-z 33277563 PMC7719161

[B76] Yang Y. Guo Y. Zhong J. Zhang T. Li D. Ba T.. (2020). Root physiological traits and transcriptome analyses reveal that root zone water retention confers drought tolerance to *Opisthopappus taihangensis* . Sci. Rep. 10, 2627. doi: 10.1038/s41598-020-59399-0 32060321 PMC7021704

[B77] Yeung E. van Veen H. Vashisht D. Sobral Paiva A. L. Hummel M. Rankenberg T.. (2018). A stress recovery signaling network for enhanced flooding tolerance in *Arabidopsis thaliana* . Proc. Natl. Acad. Sci. 115, E6085–E6094. doi: 10.1073/pnas.1803841115 29891679 PMC6042063

[B78] Yulia A. E. Sitorus J. (2012). Growth response of palm oil (Elaeis guineensis Jacq.) seedling in peat media with different flooding periods. Jurnal Agroteknologi Tropika 1, 14–17.

[B79] Zandalinas S. I. Fichman Y. Devireddy A. R. Sengupta S. Azad R. K. Mittler R. (2020). Systemic signaling during abiotic stress combination in plants. Proc. Natl. Acad. Sci. 117, 13810–13820. doi: 10.1073/pnas.2005077117 32471943 PMC7306788

[B80] Zhou L. Yarra R. (2021). Genome-wide identification and characterization of *AP2/ERF* transcription factor family genes in oil palm under abiotic stress conditions. Int. J. Mol. Sci. 22, 2821. doi: 10.3390/ijms22062821 33802225 PMC8000548

