# Supplementary material for: Transcriptomic responses of oil palm (Elaeis guineensis) stem to waterlogging at plantation in relation to precipitation seasonality
Source: Front Plant Sci. 2023 Aug 10;14:1213496. doi: 10.3389/fpls.2023.1213496 (PMC10448820; doi:10.3389/fpls.2023.1213496)
Supplement: Supplementary file 1 [file DataSheet_1.pdf]

Supplementary Material

Transcriptomic responses of oil palm (*Elaeis guineensis*) to waterlogging at plantation in relation to precipitation seasonality

Hui Lim[1], Masaki J. Kobayashi[2], Sri Nugroho Marsoem[3], Denny Irawati[3], Akihiko Kosugi[4,5], Toshiaki Kondo[4], Naoki Tani[2,5]*

*** Correspondence:** Naoki Tani: ntani@affrc.go.jp

1 Supplementary Data

Supplementary data 1: Enriched GO and KEGG terms of PDEGs and NDEGs in the oil palm stem core samples.

Supplementary data 2: Lists of overlapped DEGs identified, enriched GO and KEGG terms of overlapped DEGs in the oil palm stem core (No result obtained for enriched GO terms of overlapped NDEGs).

2 Supplementary Figures and Tables

2.1 Supplementary Figures

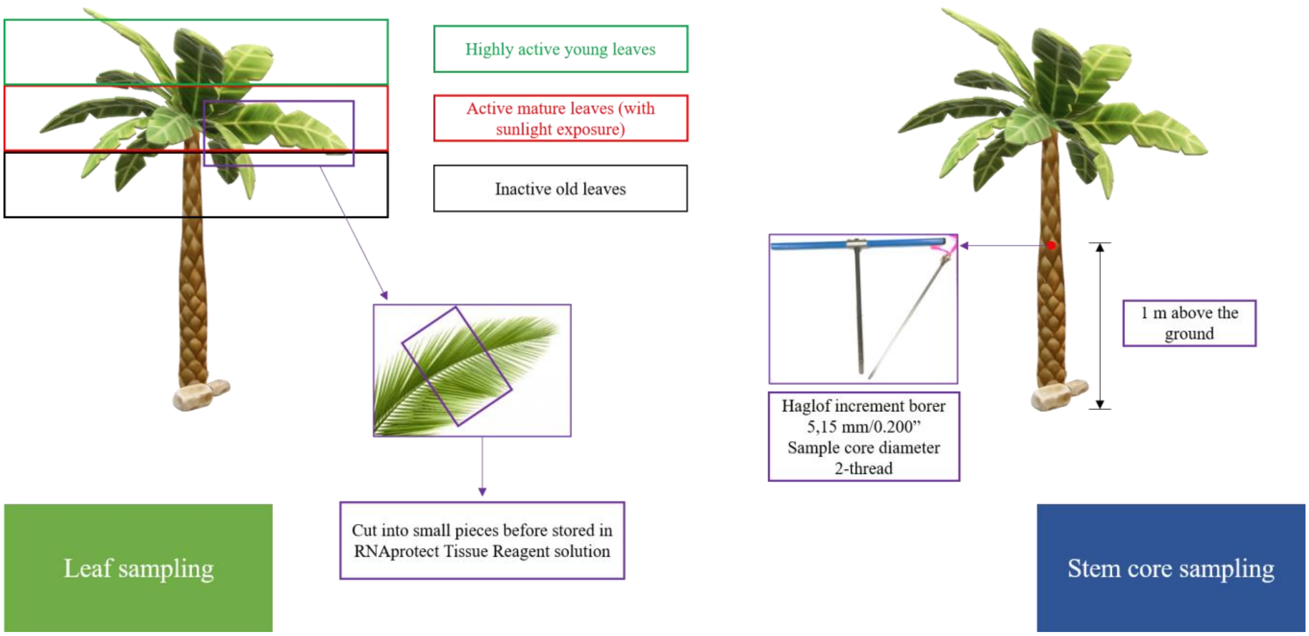

Figure S1: An illustration figure of samplings performed on adult oil palms' leaves and stem cores.

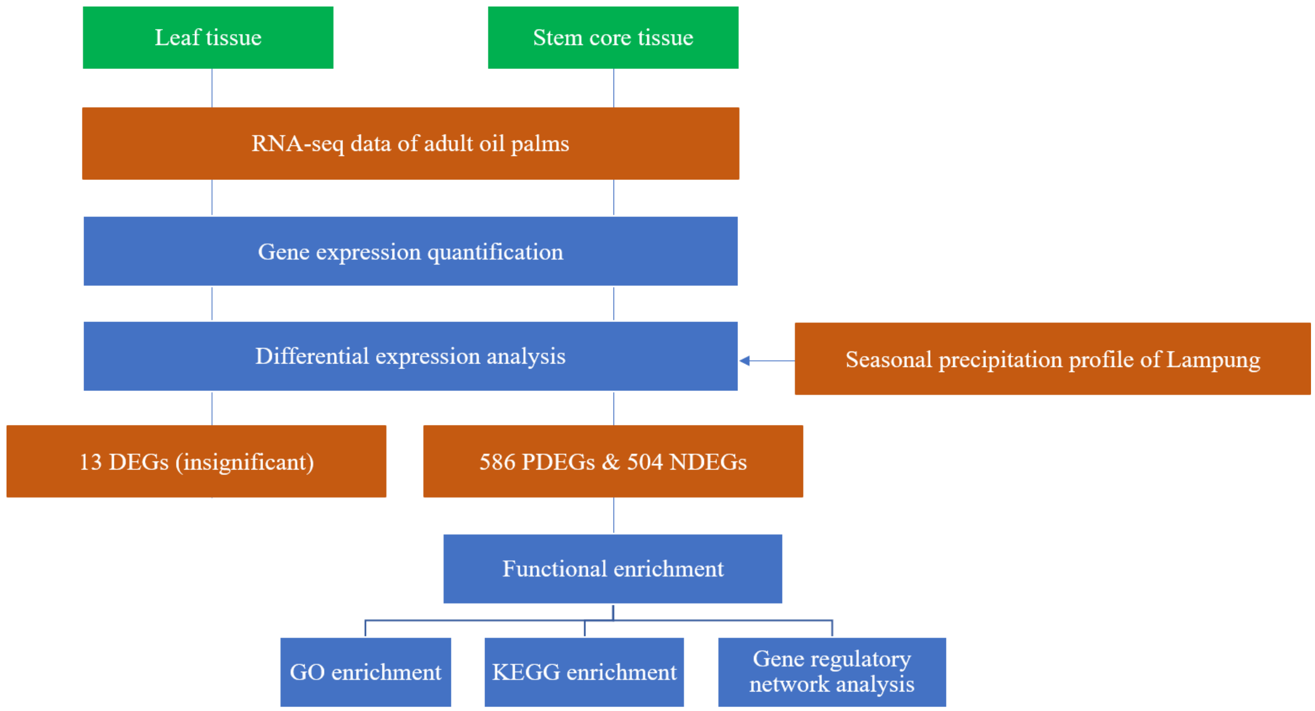

Figure S2: A workflow of RNA-seq analysis conducted in this study. Green boxes represent sampled plant organs; orange boxes represent data used to be analyzed in this study; blue boxes indicate analysis methods to process the data.

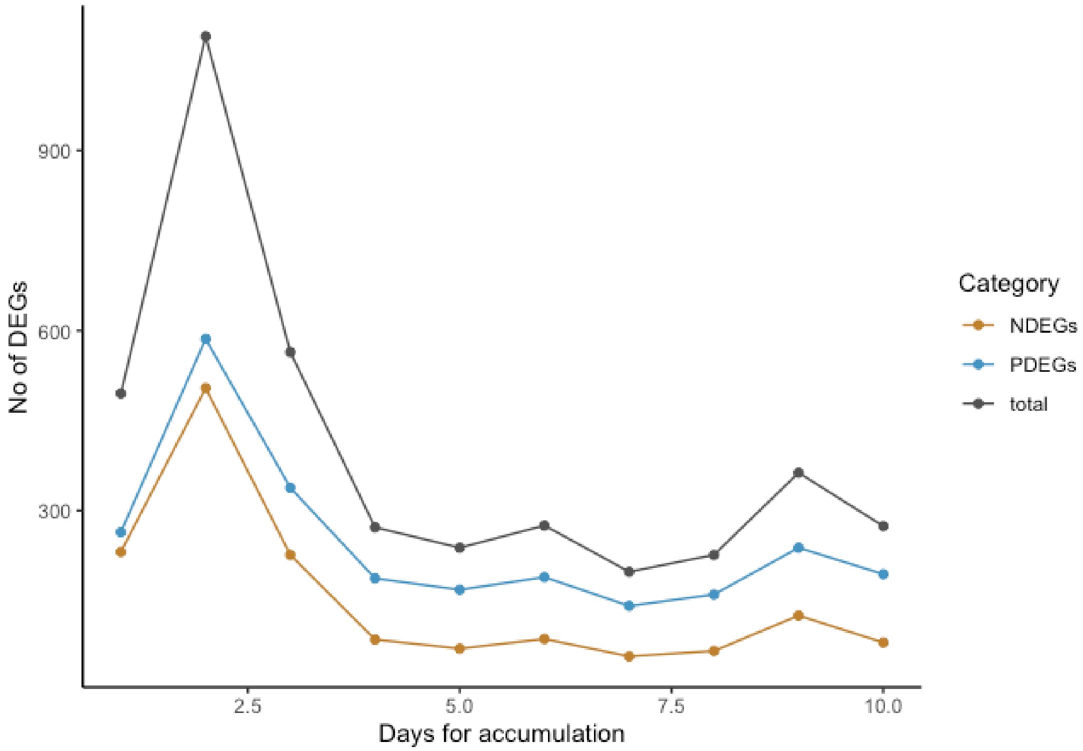

Figure S3: Numbers of positively correlated differentially expressed genes (PDEGs) (blue line), negatively correlated DEGs (NDEGs) (orange line), and total DEGs (black line) identified in the stem core by correlating transcriptomic data with the Lampung precipitation data aggregated over periods of 1-10 days.

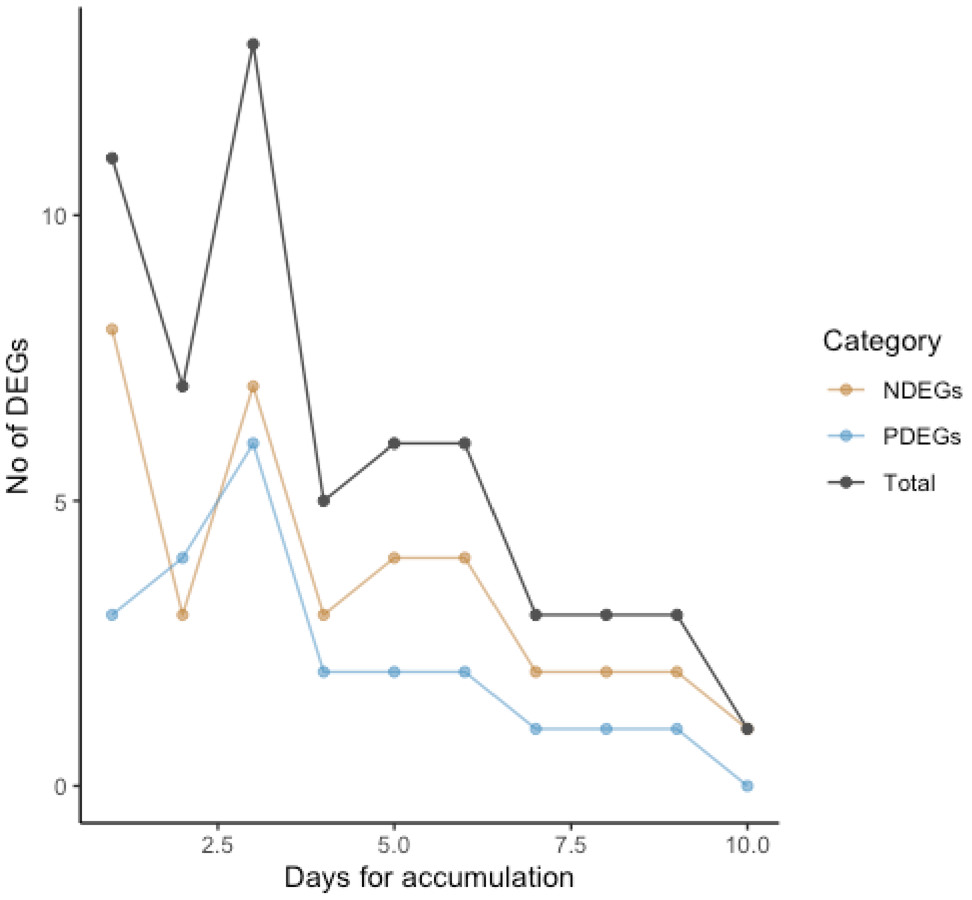

Figure S4: Numbers of positively correlated differentially expressed genes (PDEGs) (blue line), negatively correlated DEGs (NDEGs) (orange line), and total DEGs (black line) identified in the leaf samples by correlating transcriptomic data with the Lampung precipitation data aggregated over periods of 1-10 days.

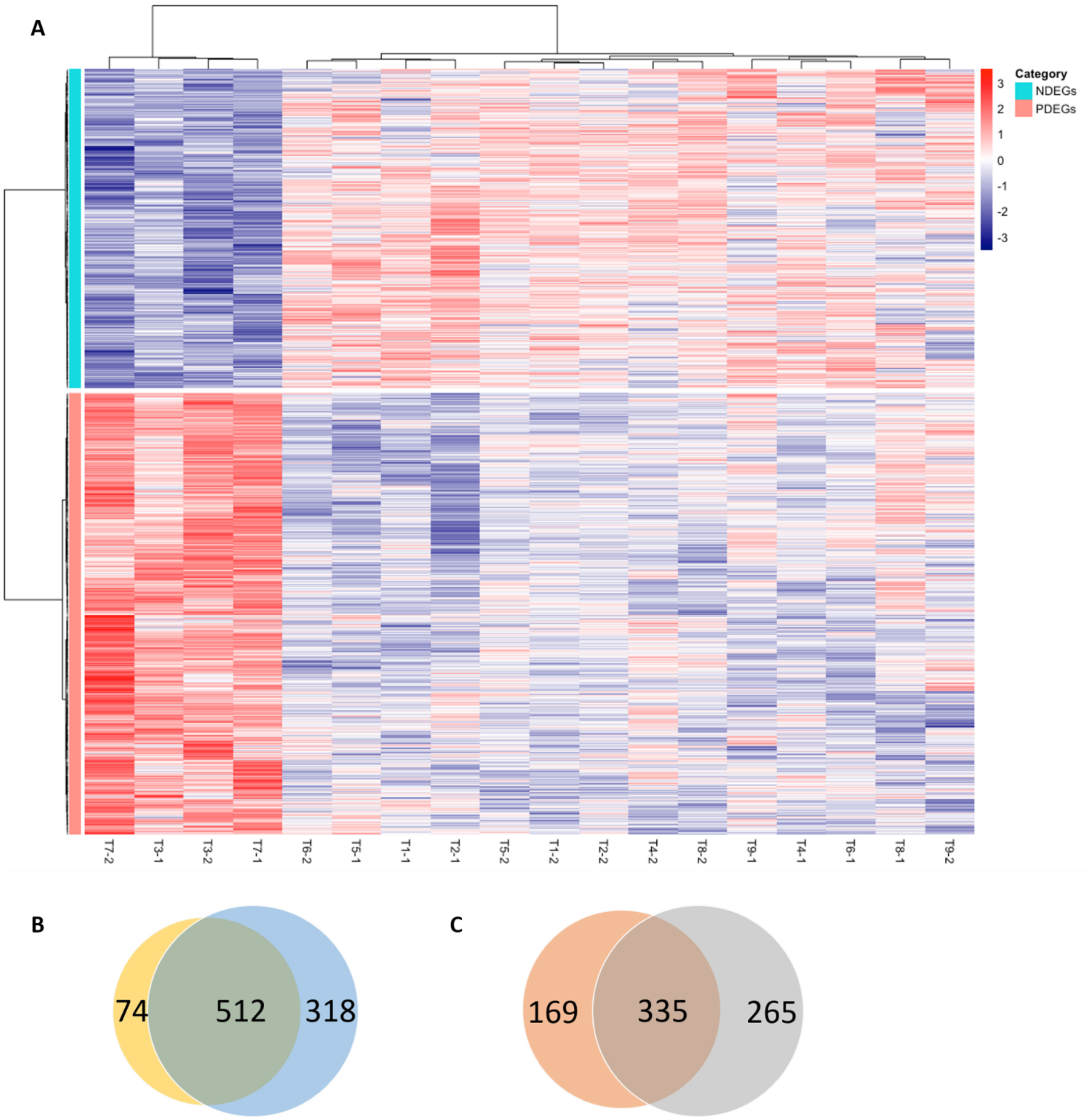

Figure S5: (A) Stem core transcriptomic expression patterns of mature oil palms at the Lampung plantation by grouping T3 and T7 as a waterlogging group for comparison. The red and purple colour scales represent the relative expression levels from higher to lower expression. PDEGs (green) and NDEGs (light-blue) are genes exhibiting positive and negative differential expression, respectively. The labels TX–1 and TX–2 denote the two samples collected at time point TX. (B) Overlapped PDEGs (512) between PDEGs obtained from expression data of correlated to 2-days precipitation accumulation (yellow, 586) and PDEGs obtained from expression data by grouping T3 and T7 together and compared to another group which consists of other sampling timepoints (blue, 830). (C) Overlapped NDEGs (335) between NDEGs obtained from expression data of correlated to 2-days

precipitation accumulation (orange, 504) and NDEGs obtained from expression data by grouping T3 and T7 together and compared to another group which consists of other sampling timepoints (grey, 600).

```
>XP_010931684.1 ERF071 LOC105052544 (HRE2)
MCGGAIISDLLPPRSQHHQLVATSDLWPGPGTDPTQKEPKKRERKNLYRGIRRRPWGKWAAEIRDPAKGVRVWLGTFSTA
EEAARAYDRAARRIRGCKAKVNFPNEDPPPPPAKAARQRYGPYCVGGEAYSSAMVAVGGGVEAEGLRQEAMASGSCADLL
EMPFVEVTAAAQAGASAGGQKGGPASNEDLFWNFEDLLF

MCGGAI: a conserved MC motif
```

Figure S6: A conserved MC motif found in the HRE2 protein (LOC105052544). The conserved MC motif is highlighted in red.
